# Supplementary material for: Identification and genomic characterization of BA.3.2: a highly divergent BA.3-related SARS-CoV-2 lineage from southern Africa
Source: Virus Evol. 2026 Mar 18;12(1):veag016. doi: 10.1093/ve/veag016 (PMC13070381; doi:10.1093/ve/veag016)
Supplement: Supplementary_materials_veag016 [file supplementary_materials_veag016.pdf]

Supplementary Information for:

Identification and genomic characterisation of BA.3.2: a highly divergent BA.3-related SARS-CoV-2 lineage from southern Africa

Graeme Dor[1], Dikeledi Kekana[2], Ryan Hisner[3], Darren P. Martin[3], Bette Korber[4,5], Timo Ernst[6,7], Avram Levy[6,7], David Speers[6,8], Stuart Turville[9], Vitali Sintchenko[10], Kerri Basile[10], Geraldine Sullivan[10], Rebecca Rockett[10], Jen Kok[10], Josette Schoenmakers[11], Federico Gueli[12], Richard Lessels[13], Cheryl Baxter[1], Nicole Wolter[2,14], Anne von Gottberg[2,14], Houriiyah Tegally[1], Tulio de Oliveira[1,13]

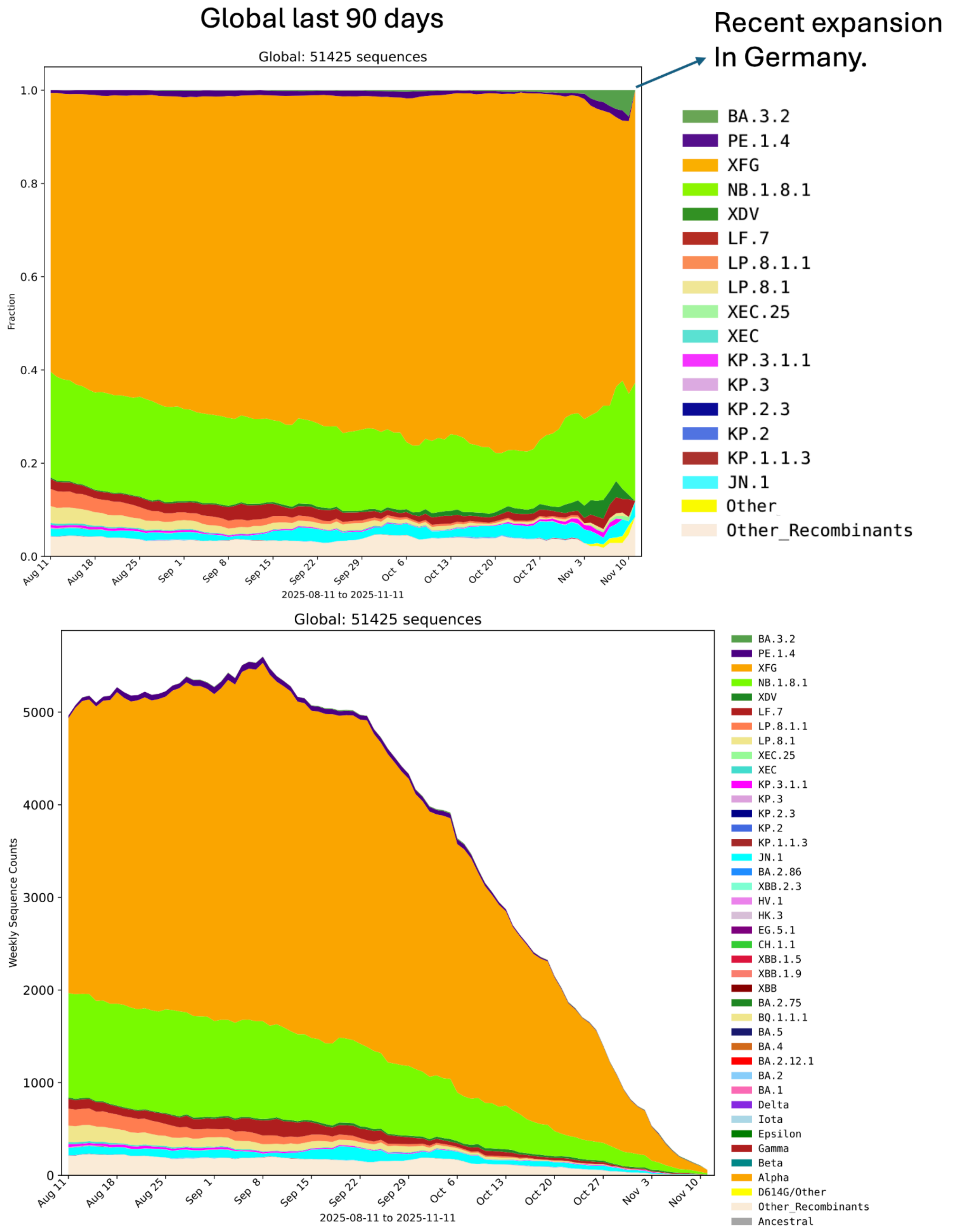

Supplementary Figure S1 - Global lineage proportion and volume of SARS-CoV-2 genomes sequenced over the most recent months, highlighting the recent increase in proportion of sequences attributed to BA.3.2

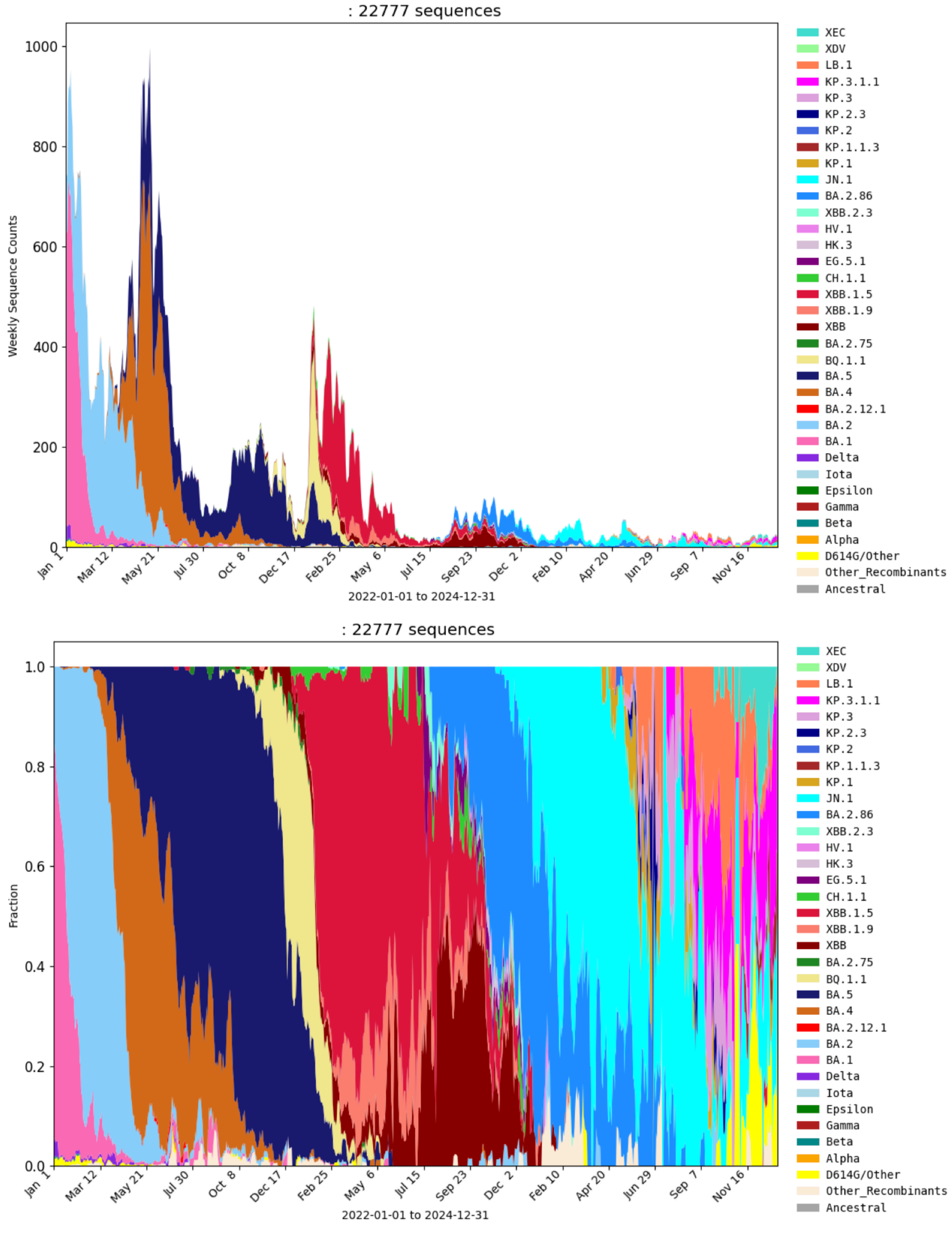

Supplementary Figure S2. **Lineage volume and proportion of SARS-CoV-2 genomes sequenced in South Africa between 2022 and 2025**, highlighting continued albeit reduced genomic surveillance during the period in which intermediate BA.3-related lineages were not detected.

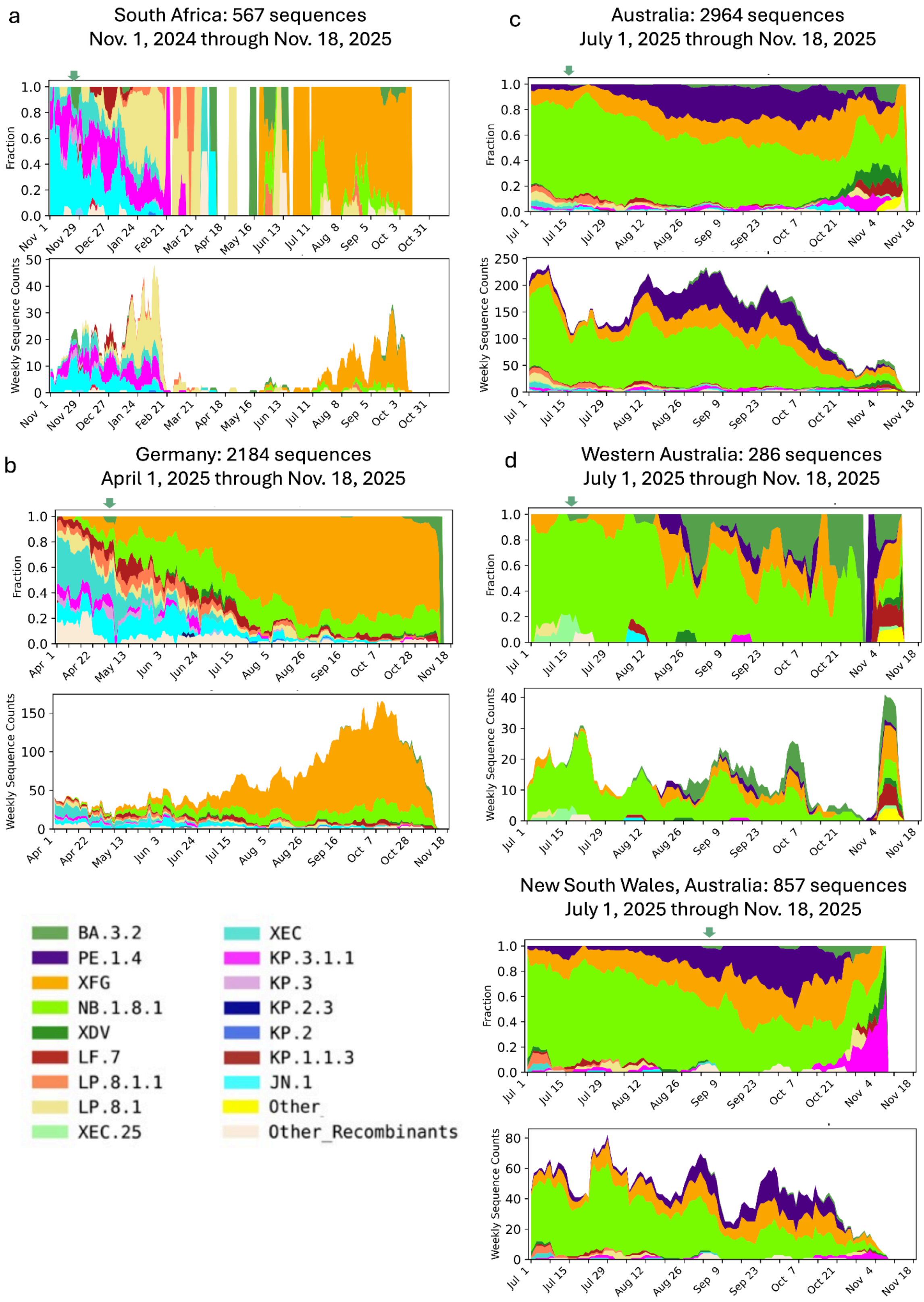

a
South Africa: 567 sequences
Nov. 1, 2024 through Nov. 18, 2025
b
Germany: 2184 sequences
April 1, 2025 through Nov. 18, 2025
c
Australia: 2964 sequences
July 1, 2025 through Nov. 18, 2025
d
Western Australia: 286 sequences
July 1, 2025 through Nov. 18, 2025
New South Wales, Australia: 857 sequences
July 1, 2025 through Nov. 18, 2025
Fraction
Weekly Sequence Counts
BA.3.2
PE.1.4
XFG
NB.1.8.1
XDV
LF.7
LP.8.1.1
LP.8.1
XEC.25
XEC
KP.3.1.1
KP.3
KP.2.3
KP.2
KP.1.1.3
JN.1
Other
Other_Recombinants

Supplementary Figure S3. **Plots illustrating the frequency of BA.3.2 lineages sampled in the three countries where BA.3.2 has been most commonly detected**. Plots were created using the Geographically Restricted Embers tool at the Los Alamos COVID-19 Viral Genome Pipeline (Korber et al. 2020) (https://cov.lanl.gov/content/sequence/EMBERS/embers.html) to illustrate the weekly frequency (top) and sequence counts (bottom) of Pango lineage designations over time that were assigned to GISAID (Khare et al. 2021) data using UShER (McBroome et al. 2021). Each plot starts with the month BA.3.2 was first detected in a given country and includes all GISAID data available through Nov. 20, 2025 (the last sampling date was Nov. 18 in this set). Pango sub-lineages are grouped into the major lineage designations to enable visualization, for example BA.3.2 is the top lineage in each plot, labeled in green, and includes the BA.3.2.1 and BA.3.2.2 sublineages. The week BA.3.2 was first detected is highlighted with a small green arrow over the "fraction" plots. (a) South African SARS-CoV-2 lineage frequencies. (b) German SARS-CoV-2 lineage frequencies. (c) Australian SARS-CoV-2 lineage frequencies. (d) SARS-CoV-2 lineage frequencies in the two states in Australia where BA.3.2 has been detected as of this sampling.

Supplementary Table S1 - Cov-Spectrum search queries

Description	Search
non-Omicron, non-recombinant sequences with S:D405N	Advanced Filters — Sequence quality: 0 ≤ Mixed sites score ≤ 101, SNP clusters score ≤ 51, Frame shifts score ≤ 51, Stop codons score ≤ 51, 0.8 ≤ Coverage (from 0 to 1)<br><br>Date Range: 2020-01-06 to 2022-01-01<br><br>S:D405N & [exactly-0-of: ins_S:214:EPE, Nextcladepangolineage:B.1.1.529*, Nextcladepangolineage:XA*, Nextcladepangolineage:XB*, Nextcladepangolineage:XC*, Nextcladepangolineage:XD*, Nextcladepangolineage:XE*, Nextcladepangolineage:XF*, Nextcladepangolineage:XG*, Nextcladepangolineage:XH*, Nextcladepangolineage:XJ*, Nextcladepangolineage:XK*, Nextcladepangolineage:XL*, Nextcladepangolineage:XM*, Nextcladepangolineage:XN*, Nextcladepangolineage:XP*, Nextcladepangolineage:XQ*, Nextcladepangolineage:XR*, Nextcladepangolineage:XS*, Nextcladepangolineage:XT*, Nextcladepangolineage:XU*, Nextcladepangolineage:XV*, Nextcladepangolineage:XW*, Nextcladepangolineage:XY*, Nextcladepangolineage:XZ*, Nextcladepangolineage:XAA*, Nextcladepangolineage:XAB*, Nextcladepangolineage:XAC*, Nextcladepangolineage:XAD*, Nextcladepangolineage:XAE*, Nextcladepangolineage:XAF*, Nextcladepangolineage:XAG*, Nextcladepangolineage:XAH*, Nextcladepangolineage:XAJ*, Nextcladepangolineage:XAK*, Nextcladepangolineage:XAL*, Nextcladepangolineage:XAM*, Nextcladepangolineage:XAN*, Nextcladepangolineage:XAP*, Nextcladepangolineage:XAQ*, Nextcladepangolineage:XAR*, Nextcladepangolineage:XAS*, Nextcladepangolineage:XAT*, Nextcladepangolineage:XAU*, Nextcladepangolineage:XAV*, Nextcladepangolineage:XAW*, Nextcladepangolineage:XAY*, Nextcladepangolineage:XAZ*, Nextcladepangolineage:XBA*, Nextcladepangolineage:XBB*, Nextcladepangolineage:XBC*, Nextcladepangolineage:XBD*, Nextcladepangolineage:XBE*, Nextcladepangolineage:XBF*, Nextcladepangolineage:XBG*, Nextcladepangolineage:XBH*, Nextcladepangolineage:XBJ*, Nextcladepangolineage:XBK*, Nextcladepangolineage:XBL*, Nextcladepangolineage:XBM*, Nextcladepangolineage:XBN*, Nextcladepangolineage:XBP*, Nextcladepangolineage:XBQ*, Nextcladepangolineage:XBR*, Nextcladepangolineage:XBS*, Nextcladepangolineage:XBT*, Nextcladepangolineage:XBU*, Nextcladepangolineage:XBV*, Nextcladepangolineage:XBW*, Nextcladepangolineage:XBY*, Nextcladepangolineage:XBZ*, Nextcladepangolineage:XCA*, Nextcladepangolineage:XCB*, Nextcladepangolineage:XCC*, Nextcladepangolineage:XCD*, Nextcladepangolineage:XCE*, Nextcladepangolineage:XCF*, Nextcladepangolineage:XCG*, Nextcladepangolineage:XCH*, Nextcladepangolineage:XCJ*, Nextcladepangolineage:XCK*, Nextcladepangolineage:XCL*, Nextcladepangolineage:XCM*, Nextcladepangolineage:XCN*, Nextcladepangolineage:XCP*, Nextcladepangolineage:XCQ*, Nextcladepangolineage:XCR*, Nextcladepangolineage:XCS*, Nextcladepangolineage:XCT*, Nextcladepangolineage:XCU*, Nextcladepangolineage:XCV*, Nextcladepangolineage:XCW*, Nextcladepangolineage:XCY*, Nextcladepangolineage:XCZ*, Nextcladepangolineage:XDA*, Nextcladepangolineage:XDB*, Nextcladepangolineage:XDC*, Nextcladepangolineage:XDD*, Nextcladepangolineage:XDE*, Nextcladepangolineage:XDF*, Nextcladepangolineage:XDG*, Nextcladepangolineage:XDH*, Nextcladepangolineage:XDJ*, Nextcladepangolineage:XDK*, Nextcladepangolineage:XDL*, Nextcladepangolineage:XDM*, Nextcladepangolineage:XDN*, Nextcladepangolineage:XDP*, Nextcladepangolineage:XDQ*, Nextcladepangolineage:XDR*, Nextcladepangolineage:XDS*, Nextcladepangolineage:XDT*, Nextcladepangolineage:XDU*, Nextcladepangolineage:XDV*, Nextcladepangolineage:XDW*, Nextcladepangolineage:XDY*, Nextcladepangolineage:XDZ*, Nextcladepangolineage:XEA*, Nextcladepangolineage:XEB*, Nextcladepangolineage:XEC*, Nextcladepangolineage:XED*, Nextcladepangolineage:XEE*, Nextcladepangolineage:XEF*, Nextcladepangolineage:XEG*, Nextcladepangolineage:XEH*, Nextcladepangolineage:XEJ*, Nextcladepangolineage:XEK*, Nextcladepangolineage:XEL*, Nextcladepangolineage:XEM*, Nextcladepangolineage:XEN*, Nextcladepangolineage:XEP*, Nextcladepangolineage:XEQ*, Nextcladepangolineage:XER*, Nextcladepangolineage:XES*, Nextcladepangolineage:XET*, Nextcladepangolineage:XEU*, Nextcladepangolineage:XEV*, Nextcladepangolineage:XEW*, Nextcladepangolineage:XEY*, Nextcladepangolineage:XEZ*, Nextcladepangolineage:XFA*, Nextcladepangolineage:XFB*, Nextcladepangolineage:XFC*, Nextcladepangolineage:XFD*, Nextcladepangolineage:XFE*, Nextcladepangolineage:XFF*, Nextcladepangolineage:XFG*, Nextcladepangolineage:XFH*, Nextcladepangolineage:XFJ*, Nextcladepangolineage:XFK*, Nextcladepangolineage:XFL*, Nextcladepangolineage:XFM*, Nextcladepangolineage:XFN*, Nextcladepangolineage:XFP*, Nextcladepangolineage:XFQ*, Nextcladepangolineage:XFR*, Nextcladepangolineage:XFS*, Nextcladepangolineage:XFT*, Nextcladepangolineage:XFU*, Nextcladepangolineage:XFV*, Nextcladepangolineage:XFW*, Nextcladepangolineage:XFY*, Nextcladepangolineage:XFZ*, Nextcladepangolineage:XGA*, Nextcladepangolineage:XGB*, [5-of: G10447A, A27259C, S:V213G, S:G339D, S:S371F, S:S371L, S:S373P, S:S375F, S:T376A, S:R408S, S:K417N, S:N440K, S:S477N, S:T478K, S:E484A, S:Q493R, S:Q498R, S:N679K, S:N764K, S:D796Y, S:N856K, S:Q954H, S:N969K, S:L981F, M:Q19E, N:S413R, ORF3a:T223I, ORF6:D61L, E:T9I, ORF1a:L3027F, ORF1a:T3090I, ORF1a:P3395H, ORF1b:I1566V, ORF1b:T2163I]]

non-Omicron, non-recombinant sequences with R408S	Advanced Filters — Sequence quality: 0 ≤ Mixed sites score ≤ 101, SNP clusters score ≤ 51, Frame shifts score ≤ 51, Stop codons score ≤ 51, 0.8 ≤ Coverage (from 0 to 1)<br><br>S:R408S & [exactly-0-of: ins_S:214:EPE, C22792T, Nextcladepangolineage:B.1.1.529*, Nextcladepangolineage:XA*, Nextcladepangolineage:XB*, Nextcladepangolineage:XC*, Nextcladepangolineage:XD*, Nextcladepangolineage:XE*, Nextcladepangolineage:XF*, Nextcladepangolineage:XG*, Nextcladepangolineage:XH*, Nextcladepangolineage:XJ*, Nextcladepangolineage:XK*, Nextcladepangolineage:XL*, Nextcladepangolineage:XM*, Nextcladepangolineage:XN*, Nextcladepangolineage:XP*, Nextcladepangolineage:XQ*, Nextcladepangolineage:XR*, Nextcladepangolineage:XS*, Nextcladepangolineage:XT*, Nextcladepangolineage:XU*, Nextcladepangolineage:XV*, Nextcladepangolineage:XW*, Nextcladepangolineage:XY*, Nextcladepangolineage:XZ*, Nextcladepangolineage:XAA*, Nextcladepangolineage:XAB*, Nextcladepangolineage:XAC*, Nextcladepangolineage:XAD*, Nextcladepangolineage:XAE*, Nextcladepangolineage:XAF*, Nextcladepangolineage:XAG*, Nextcladepangolineage:XAH*, Nextcladepangolineage:XAJ*, Nextcladepangolineage:XAK*, Nextcladepangolineage:XAL*, Nextcladepangolineage:XAM*, Nextcladepangolineage:XAN*, Nextcladepangolineage:XAP*, Nextcladepangolineage:XAQ*, Nextcladepangolineage:XAR*, Nextcladepangolineage:XAS*, Nextcladepangolineage:XAT*, Nextcladepangolineage:XAU*, Nextcladepangolineage:XAV*, Nextcladepangolineage:XAW*, Nextcladepangolineage:XAY*, Nextcladepangolineage:XAZ*, Nextcladepangolineage:XBA*, Nextcladepangolineage:XBB*, Nextcladepangolineage:XBC*, Nextcladepangolineage:XBD*, Nextcladepangolineage:XBE*, Nextcladepangolineage:XBF*, Nextcladepangolineage:XBG*, Nextcladepangolineage:XBH*, Nextcladepangolineage:XBJ*, Nextcladepangolineage:XBK*, Nextcladepangolineage:XBL*, Nextcladepangolineage:XBM*, Nextcladepangolineage:XBN*, Nextcladepangolineage:XBP*, Nextcladepangolineage:XBQ*, Nextcladepangolineage:XBR*, Nextcladepangolineage:XBS*, Nextcladepangolineage:XBT*, Nextcladepangolineage:XBU*, Nextcladepangolineage:XBV*, Nextcladepangolineage:XBW*, Nextcladepangolineage:XBY*, Nextcladepangolineage:XBZ*, Nextcladepangolineage:XCA*, Nextcladepangolineage:XCB*, Nextcladepangolineage:XCC*, Nextcladepangolineage:XCD*, Nextcladepangolineage:XCE*, Nextcladepangolineage:XCF*, Nextcladepangolineage:XCG*, Nextcladepangolineage:XCH*, Nextcladepangolineage:XCJ*, Nextcladepangolineage:XCK*, Nextcladepangolineage:XCL*, Nextcladepangolineage:XCM*, Nextcladepangolineage:XCN*, Nextcladepangolineage:XCP*, Nextcladepangolineage:XCQ*, Nextcladepangolineage:XCR*, Nextcladepangolineage:XCS*, Nextcladepangolineage:XCT*, Nextcladepangolineage:XCU*, Nextcladepangolineage:XCV*, Nextcladepangolineage:XCW*, Nextcladepangolineage:XCY*, Nextcladepangolineage:XCZ*, Nextcladepangolineage:XDA*, Nextcladepangolineage:XDB*, Nextcladepangolineage:XDC*, Nextcladepangolineage:XDD*, Nextcladepangolineage:XDE*, Nextcladepangolineage:XDF*, Nextcladepangolineage:XDG*, Nextcladepangolineage:XDH*, Nextcladepangolineage:XDJ*, Nextcladepangolineage:XDK*, Nextcladepangolineage:XDL*, Nextcladepangolineage:XDM*, Nextcladepangolineage:XDN*, Nextcladepangolineage:XDP*, Nextcladepangolineage:XDQ*, Nextcladepangolineage:XDR*, Nextcladepangolineage:XDS*, Nextcladepangolineage:XDT*, Nextcladepangolineage:XDU*, Nextcladepangolineage:XDV*, Nextcladepangolineage:XDW*, Nextcladepangolineage:XDY*, Nextcladepangolineage:XDZ*, Nextcladepangolineage:XEA*, Nextcladepangolineage:XEB*, Nextcladepangolineage:XEC*, Nextcladepangolineage:XED*, Nextcladepangolineage:XEE*, Nextcladepangolineage:XEF*, Nextcladepangolineage:XEG*, Nextcladepangolineage:XEH*, Nextcladepangolineage:XEJ*, Nextcladepangolineage:XEK*, Nextcladepangolineage:XEL*, Nextcladepangolineage:XEM*, Nextcladepangolineage:XEN*, Nextcladepangolineage:XEP*, Nextcladepangolineage:XEQ*, Nextcladepangolineage:XER*, Nextcladepangolineage:XES*, Nextcladepangolineage:XET*, Nextcladepangolineage:XEU*, Nextcladepangolineage:XEV*, Nextcladepangolineage:XEW*, Nextcladepangolineage:XEY*, Nextcladepangolineage:XEZ*, Nextcladepangolineage:XFA*, Nextcladepangolineage:XFB*, Nextcladepangolineage:XFC*, Nextcladepangolineage:XFD*, Nextcladepangolineage:XFE*, Nextcladepangolineage:XFF*, Nextcladepangolineage:XFG*, Nextcladepangolineage:XFH*, Nextcladepangolineage:XFJ*, Nextcladepangolineage:XFK*, Nextcladepangolineage:XFL*, Nextcladepangolineage:XFM*, Nextcladepangolineage:XFN*, Nextcladepangolineage:XFP*, Nextcladepangolineage:XFQ*, Nextcladepangolineage:XFR*, Nextcladepangolineage:XFS*, Nextcladepangolineage:XFT*, Nextcladepangolineage:XFU*, Nextcladepangolineage:XFV*, Nextcladepangolineage:XFW*, Nextcladepangolineage:XFY*, Nextcladepangolineage:XFZ*, Nextcladepangolineage:XGA*, Nextcladepangolineage:XGB*, [5-of: G10447A, A27259C, S:V213G, S:G339D, S:S371F, S:S371L, S:S373P, S:S375F, S:T376A, S:D405N, S:K417N, S:N440K, S:S477N, S:T478K, S:E484A, S:Q493R, S:Q498R, S:N679K, S:N764K, S:D796Y, S:N856K, S:Q954H, S:N969K, S:L981F, M:Q19E, N:S413R, ORF3a:T223I, ORF6:D61L, E:T9I, ORF1a:L3027F, ORF1a:T3090I, ORF1a:P3395H, ORF1b:I1566V, ORF1b:T2163I]]
BA.1* with R408S and not S:D405N	Nextcladepangolineage:BA.1* & R408S & [54-of: S:S371L, S:S373P, S:S375F, S:T376T, S:Y380Y, S:G381G, S:V382V, S:S383S, S:K386K, S:L387L, S:N388N, S:D389D, S:L390L, S:C391C, S:F392F, S:T393T, S:N394N, S:V395V, S:Y396Y, S:A397A, S:D398D, S:S399S, S:F400F, S:V401V, S:I402I, S:G404G, S:D405D, S:E406E, S:V407V, S:Q409Q, S:I410I, S:A411A, S:P412P, S:G413G, S:Q414Q, S:T415T, S:G416G, S:K417N, S:I418I, S:A419A, S:Y421Y, S:N422N, S:Y423Y, S:K424K, S:L425L, S:P426P, S:D427D, S:D428D, S:F429F, S:G431G, S:C432C, S:V433V, S:N440K, S:G446S] & [exactly-0-of: T670G, C2790T, G4184A, C4321T, C9344T, A9424G, C9534T, C9866T, C10198T, G10447A, C12880T, C15714T, C17410T, C19955T, A20055G, C21618T, T21633-, A21634-, C21635-, C21636-, C21637-, C21638-, C21639-, T21640-, G21641-, G21987A, T22200G, C26060T, C26858T, G27382C, A27383T, T27384C, A29510C] & [exactly-0-of: T670G, C2790T, G4184A, C4321T, C9344T, A9424G, C9534T, C9866T, C10198T, G10447A, C12880T, C15714T, C17410T, C19955T, A20055G, C21618T, T21633-, A21634-, C21635-, C21636-, C21637-, C21638-, C21639-, T21640-, G21641-, G21987A, T22200G, C26060T, C26858T, G27382C, A27383T, T27384C, A29510C]

BA.1* with S:D405N and not R408S	Nextcladepangolineage:BA.1* & S:D405N & [53-of: S:S371L, S:S373P, S:S375F, S:T376T, S:Y380Y, S:G381G, S:V382V, S:S383S, S:K386K, S:L387L, S:N388N, S:D389D, S:L390L, S:C391C, S:F392F, S:T393T, S:N394N, S:V395V, S:Y396Y, S:A397A, S:D398D, S:S399S, S:F400F, S:V401V, S:I402I, S:G404G, S:E406E, S:V407V, !R408S, S:Q409Q, S:I410I, S:A411A, S:P412P, S:G413G, S:Q414Q, S:T415T, S:G416G, S:K417N, S:I418I, S:A419A, S:Y421Y, S:N422N, S:Y423Y, S:K424K, S:L425L, S:P426P, S:D427D, S:D428D, S:F429F, S:G431G, S:C432C, S:V433V, S:N440K] & [exactly-0-of: T670G, C2790T, G4184A, C4321T, C9344T, A9424G, C9534T, C9866T, C10198T, G10447A, C12880T, C15714T, C17410T, C19955T, A20055G, C21618T, T21633-, A21634-, C21635-, C21636-, C21637-, C21638-, C21639-, T21640-, G21641-, G21987A, T22200G, C26060T, C26858T, G27382C, A27383T, T27384C, A29510C]
BA.1* with both S:D405N and R408S	Nextcladepangolineage:BA.1* & S:D405N & [53-of: S:S371L, S:S373P, S:S375F, S:T376T, S:Y380Y, S:G381G, S:V382V, S:S383S, S:K386K, S:L387L, S:N388N, S:D389D, S:L390L, S:C391C, S:F392F, S:T393T, S:N394N, S:V395V, S:Y396Y, S:A397A, S:D398D, S:S399S, S:F400F, S:V401V, S:I402I, S:G404G, S:E406E, S:V407V, R408S, S:Q409Q, S:I410I, S:A411A, S:P412P, S:G413G, S:Q414Q, S:T415T, S:G416G, S:K417N, S:I418I, S:A419A, S:Y421Y, S:N422N, S:Y423Y, S:K424K, S:L425L, S:P426P, S:D427D, S:D428D, S:F429F, S:G431G, S:C432C, S:V433V, S:N440K] & [exactly-0-of: T670G, C2790T, G4184A, C4321T, C9344T, A9424G, C9534T, C9866T, C10198T, G10447A, C12880T, C15714T, C17410T, C19955T, A20055G, C21618T, T21633-, A21634-, C21635-, C21636-, C21637-, C21638-, C21639-, T21640-, G21641-, G21987A, T22200G, C26060T, C26858T, G27382C, A27383T, T27384C, A29510C] & [exactly-0-of: T670G, C2790T, G4184A, C4321T, C9344T, A9424G, C9534T, C9866T, C10198T, G10447A, C12880T, C15714T, C17410T, C19955T, A20055G, C21618T, T21633-, A21634-, C21635-, C21636-, C21637-, C21638-, C21639-, T21640-, G21641-, G21987A, T22200G, C26060T, C26858T, G27382C, A27383T, T27384C, A29510C]

Supplementary Table S2 - **BA.3.2 mutations and associated fitness estimates presented by Haddox et al. (2025)**

**Mutation**	**Fitness**	**Uncertainty**	**abs_fitness_minus_uncertainty**	**Expected_Count**	**Actual_Count**
ORF1a:A591V	1.11	0.71	0.40	757.5	2522
ORF1a:K672N	-0.51	0.70	-0.19	529.6	269
ORF1a:T1881I	-0.03	0.87	-0.84	2710.9	2631
ORF1a:V2071F	-0.31	0.91	-0.60	1103.2	773
ORF1a:I3944L	0.21	0.64	-0.43	22.3	29
ORF1b:I527T	0.12	0.48	-0.36	440.9	500
S:P9L	0.83	0.56	0.27	655.5	1568
S:R21T	2.19	1.01	1.18	99.8	1376
S:P26L	-0.47	0.68	-0.21	1768.6	1063
S:I101T	-0.27	0.66	-0.40	280.2	211
S:F157S	1.06	0.49	0.58	135.5	410
S:N164K	2.48	0.58	1.90	10.2	201
S:S172F	-1.24	0.76	0.48	605.3	155
S:K187T	0.52	0.75	-0.23	26.3	47
S:P251S	-0.55	0.65	-0.10	594.0	329
S:I326V	-0.64	0.59	0.04	258.3	132
S:D339Y	0.69	0.81	-0.12	120.2	260
S:A348P	0.98	1.01	-0.03	18.3	59
S:R403K	0.46	0.59	-0.13	178.6	292
S:R408S	1.13	0.59	0.53	34.2	170
S:A435S	-0.40	0.91	-0.50	436.0	274
S:K440R	0.33	0.51	-0.18	105.5	149
S:V445A	2.48	0.48	2.00	80.6	1061
S:N460K	2.43	0.57	1.86	42.7	615
S:K478N	1.21	0.56	0.65	29.6	119
S:G496S	0.21	0.52	-0.31	302.4	377
S:K529N	-0.51	0.69	-0.17	463.1	366
S:E554D	-0.33	0.68	-0.36	454.6	370
S:E583D	1.21	0.67	0.54	1284.9	3425
S:H625R	-0.03	0.37	-0.34	278.9	271
S:N641K	0.26	0.59	-0.33	44.1	52
S:V642G	3.92	1.00	2.92	9.5	1036
S:E654K	0.60	0.50	0.10	161.9	304
S:K679R	0.48	0.48	0.01	111.1	184
S:A688D	0.07	0.68	-0.61	64.9	70

S:S704L	0.52	0.66	-0.14	2089.5	3654
S:K795T	-0.04	0.76	-0.72	30.9	30
S:S939F	0.65	0.73	-0.08	1833.8	3725
S:D1184E	-0.92	0.63	0.28	86.0	47
ORF3a:A103S	0.36	0.90	-0.54	436.0	659
E:T11A	0.91	0.66	0.25	108.7	290
N:Q241H	0.16	0.56	-0.40	47.2	61

Mutations highlighted in yellow represent positive fitness values greater than the associated uncertainty, while mutations highlighted in orange represent negative fitness values greater than the associated uncertainty. BA.3.2 mutations not included in the fitness ratings are ORF1a:R124C, S:S446D, S:L452W, S:A484K, and S:A852K.

Supplementary Table S3 - The GISAID accession numbers and Pango lineages for the nine divergent sequences with either S:K795T or S:A852K.

**GISAID Accession**	**Pango Lineage**
EPI_ISL_14832977	BA.1.1
EPI_ISL_16613482	BA.1.1
EPI_ISL_16835399	BA.1.18
EPI_ISL_17509597	BA.1
EPI_ISL_17559165	BA.1.17.2
EPI_ISL_17988396	BA.1
EPI_ISL_18365170	BA.1.1
EPI_ISL_18908924	BA.5.2.20
EPI_ISL_19860650	BQ.1.1.1

Supplementary Results S1: Evidence for epistasis between spike residues 405 and 408

This analysis supports the inference of positive epistasis between D405N and R408S described in the main text.

Here, we quantify the co-occurrence of D405N and R408S in BA.1 sequences to support the inference of epistasis described in the main text. After excluding sequences with any other BA.2 mutations (indicating contamination, coinfection, or recombination) or dropout in the spike region immediately surrounding 405, only five high-quality BA.1 sequences possessed the D405N mutation without R408S (0.0004% of all qualifying BA.1 sequences). A similar search for BA.1 sequences with R408S but without D405N returns 10 sequences (0.0008%). The number of high-quality BA.1 sequences with both D405N and R408S (and no other BA.2 mutations), on the other hand, is 20.

Supplementary Results S2: Expanded section on deletions and mutations in the NTD

Deletions and mutations in the NTD of the BA.3.2 spike likely have substantial impacts on the stability and immune evasiveness of spike (expanded)

The SARS-CoV-2 NTD is marked by five projecting loops, N1 (14-26), N2 (67-81), N3 (140-158), N4 (173-190), and N5 (241-263) (Cantoni et al. 2022). These loops are exceptionally long in the SARS-CoV-2 Wuhan reference genome compared to other sarbecoviruses (Cantoni et al. 2022; Qing and Gallagher 2023). In SARS-CoV-2 pseudoviruses, shorter NTD loops have been shown to increase infectivity and fusion activity, but at the expense of greater spike instability and premature S1 shedding, which precludes cell entry (Cantoni et al. 2022; Meng et al. 2022; Qing and Gallagher 2023). The longer NTD loops in SARS-CoV-2 likely enabled, or else were adaptations to, the furin cleavage site (FCS) insertion at the S1/S2 boundary, which is unique among known sarbecoviruses and which makes premature S1 shedding more likely.

In addition to the three deletions it inherited from BA.3 (∆69-70, ∆142-144, and ∆N211), the BA.3.2 spike has eleven more amino acid residues deleted: a 9-AA expansion of the N3-loop deletion (∆136-147) and a 2-AA deletion in the N5 loop (∆243-244). Deletions in N3, including the much more modest ∆Y144, have previously been shown to evade some classes of antibodies (Cao et al. 2023), and in the Beta variant, an N5 deletion similar to that of BA.3.2 was shown to cause a dramatic rearrangement of the N4 loop and even contributed to the evasion of some RBD-directed antibodies (Cai et al. 2021; Gobeil et al. 2021).

Besides these deletions, the BA.3.2 NTD has eight amino acid substitutions relative to BA.3: P9L, R21T, P26L, I101T, F157S, N164K, S172F, K187T, and P251S.

It is likely that the Spike expressed by BA.3.2 is also missing most of its N1 loop. While the codon sites encoding the N1 loops remain undeleted, the P9L substitution shifts the signal peptide cleavage position from residues 13-14 to residues 21-22(Yu et al. 2023), effectively

deleting most of the N1 loop. Together with ∆136-147 this erases the C15-C136 NTD disulfide bond. Rearrangement of the NTD due to removal or alteration of the C15-C136 disulfide has been shown to efficiently evade NTD-specific neutralizing antibodies (Duyvesteyn et al. 2024; McCallum et al. 2021b; McCallum et al. 2021a). Only three lineages with a deleted C15-C136 disulfide, all relatively small, have ever circulated: B.1.640 (~1200 sequences), C.1.2 (~420 sequences), and BA.2.87.1 (~10 sequences).

Similarly rare are the S172F and K187T substitutions, each of which has appeared in only ~400 of the more than 15 million SARS-CoV-2 sequences. K187T likely creates a glycan at N185 and was in the CH.1.1.18 lineage. Over half of the approximately 400 SARS-CoV-2 sequences with S172F were Delta lineages, while fewer than 100 have been found so far in Omicron lineages other than BA.3.2. Interestingly, one highly divergent HP.1.1 (an XBB.1.5 descendant) sequence (EPI_ISL_19808706) with S172F shares several additional spike elements with BA.3.2, including NTD disulfide alteration (through C15Y and W152C), addition of an N99 glycan (through an I101T mutation), ∆243-244, mutation away from S446 (S446N for the HP.1.1, S446D for BA.3.2) and the mutations E583D, V642G, and K679R. Furthermore, like BA.3.2 (which has S:A688D), it has a mutation adjacent to the FCS (T676N, which creates a glycan) that almost certainly inhibits S1/S2 cleavage. The occurrence of such a large number of otherwise uncommon mutations in two unrelated sequences may be an indication that these mutations may be interacting with one another (i.e. via positive epistasis) to yield a fitness advantage.

The addition of three new glycans on the BA.3.2 spike (promoted by I101T, K187T, and K529N), and a fourth glycan in BA.3.2.2, which adds a glycan at N354 via its K356T mutation, could represent a long-term trend in SARS-CoV-2 evolution. The BA.2.86 spike also possessed additional S1 glycans at N245 (via H245N) and N354 (via K356T) relative to previously dominant variants. Furthermore, within a year after its first appearance, a third glycan (either at N22 via T22N or N30 via ∆S31) had become universal among BA.2.86 descendants (Li et al. 2025; Liu et al. 2025), and in spring 2025 over half of all sequences had added another glycan with the R190S mutation. As population immunity grows through repeated vaccination and infection, and antibody breadth continually increases through the affinity maturation process (Kim et al. 2022; Turner et al. 2021), the addition of new S1 glycans may represent an increasingly essential immune-evasion strategy for the virus.

Data availability

All of the SARS-CoV-2 sequences analysed and presented in the supplementary are publicly accessible through the GISAID platform (https://www.gisaid.org/), using the GISAID identifier: EPI_SET_251123so (Supplementary Figure S3a); EPI_SET_251123nh (Supplementary Figure S3b); EPI_SET_251123ve (Supplementary Figure S3c and S3d).

Reference list

Cai, Y., et al. (2021), 'Structural basis for enhanced infectivity and immune evasion of SARS-CoV-2 variants', *Science,* 373 (6555), 642-48.

Cantoni, D., et al. (2022), 'Evolutionary remodelling of N-terminal domain loops fine-tunes SARS-CoV-2 spike', *EMBO Rep,* 23 (10), e54322.

Cao, Y., et al. (2023), 'Imprinted SARS-CoV-2 humoral immunity induces convergent Omicron RBD evolution', *Nature,* 614 (7948), 521-29.

Duyvesteyn, H. M. E., et al. (2024), 'Concerted deletions eliminate a neutralizing supersite in SARS-CoV-2 BA.2.87.1 spike', *Structure,* 32 (10), 1594-602 e6.

Gobeil, S. M., et al. (2021), 'Effect of natural mutations of SARS-CoV-2 on spike structure, conformation, and antigenicity', *Science,* 373 (6555).

Haddox, H. K., et al. (2025), 'The mutation rate of SARS-CoV-2 is highly variable between sites and is influenced by sequence context, genomic region, and RNA structure', *Nucleic Acids Res,* 53 (11).

Khare, Shruti, et al. (2021), 'GISAID's Role in Pandemic Response', *China CDC Weekly,* 3 (49), 1049-51.

Kim, W., et al. (2022), 'Germinal centre-driven maturation of B cell response to mRNA vaccination', *Nature,* 604 (7904), 141-45.

Korber, B., et al. (2020), 'Tracking Changes in SARS-CoV-2 Spike: Evidence that D614G Increases Infectivity of the COVID-19 Virus', *Cell,* 182 (4), 812-27 e19.

Li, P., et al. (2025), 'Role of glycosylation mutations at the N-terminal domain of SARS-CoV-2 XEC variant in immune evasion, cell-cell fusion, and spike stability', *J Virol,* 99 (4), e0024225.

Liu, J., et al. (2025), 'Enhanced immune evasion of SARS-CoV-2 variants KP.3.1.1 and XEC through N-terminal domain mutations', *Lancet Infect Dis,* 25 (1), e6-e7.

McBroome, J., et al. (2021), 'A Daily-Updated Database and Tools for Comprehensive SARS-CoV-2 Mutation-Annotated Trees', *Mol Biol Evol,* 38 (12), 5819-24.

McCallum, M., et al. (2021a), 'SARS-CoV-2 immune evasion by the B.1.427/B.1.429 variant of concern', *Science,* 373 (6555), 648-54.

--- (2021b), 'N-terminal domain antigenic mapping reveals a site of vulnerability for SARS-CoV-2', *Cell,* 184 (9), 2332-47 e16.

Meng, B., et al. (2022), 'SARS-CoV-2 spike N-terminal domain modulates TMPRSS2-dependent viral entry and fusogenicity', *Cell Rep,* 40 (7), 111220.

Qing, E. and Gallagher, T. (2023), 'Adaptive variations in SARS-CoV-2 spike proteins: effects on distinct virus-cell entry stages', *mBio,* 14 (4), e0017123.

Turner, J. S., et al. (2021), 'SARS-CoV-2 infection induces long-lived bone marrow plasma cells in humans', *Nature,* 595 (7867), 421-25.

Yu, X., et al. (2023), 'Convergence of immune escape strategies highlights plasticity of SARS-CoV-2 spike', *PLoS Pathog,* 19 (5), e1011308.
